# Supplementary material for: Comprehensive Annotation and Functional Exploration of MicroRNAs in Lettuce
Source: Front Plant Sci. 2021 Dec 24;12:781836. doi: 10.3389/fpls.2021.781836 (PMC8739914; doi:10.3389/fpls.2021.781836)
Supplement: Supplementary file 2 [file Data_Sheet_8.ZIP › GSM2230749.plot/Lsa-miRN1690_Lsat_1_v5_gn_5_159861.1_62_TPlot.pdf]

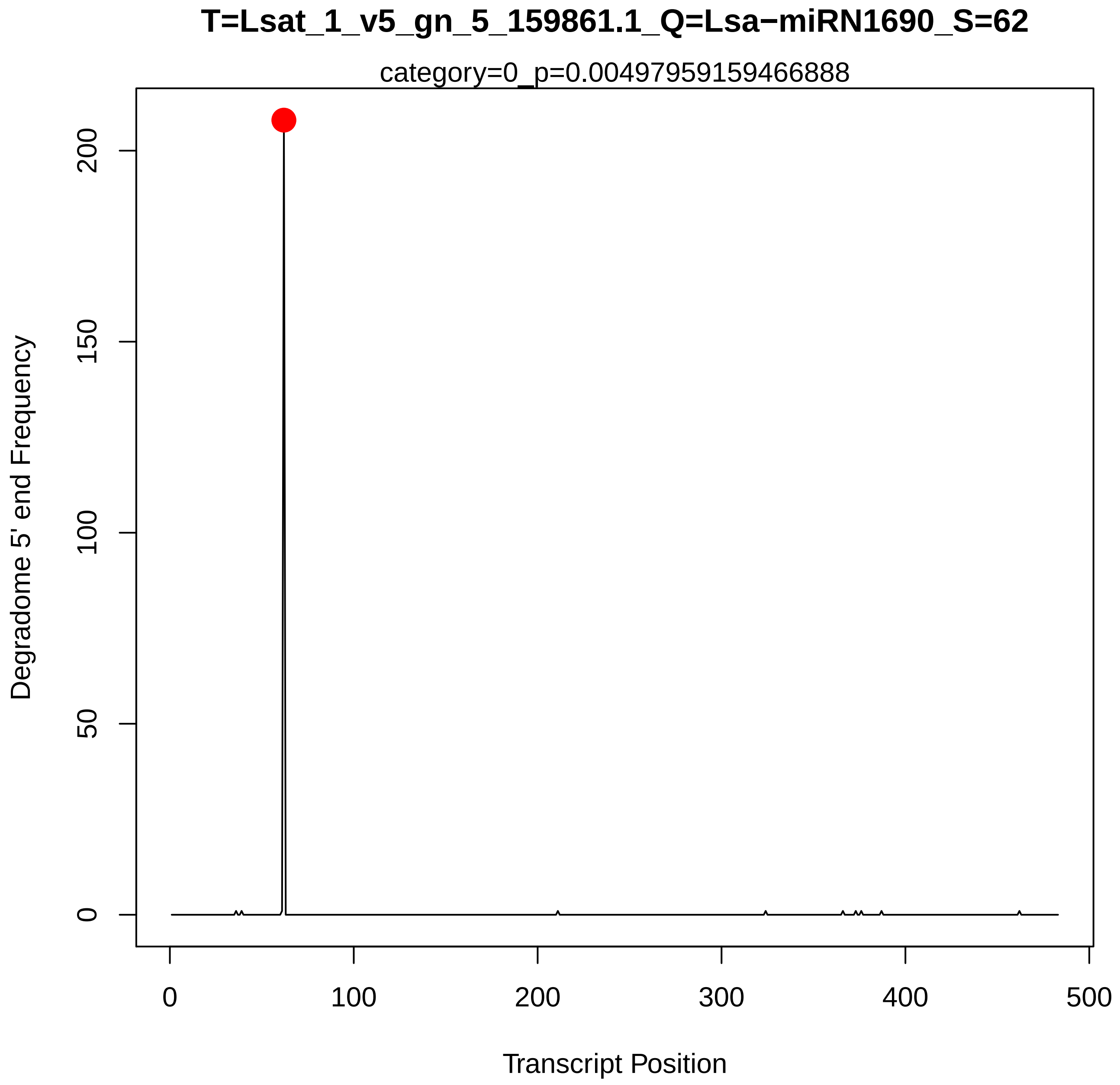

T=Lsat_1_v5_gn_5_159861.1_Q=Lsa−miRN1690_S=62
category=0_p=0.00497959159466888
Degradome 5' end Frequency
0
50
100
150
200
0
100
200
300
400
500
Transcript Position
